# Age-related differences in diabetes care outcomes in Korea: a retrospective cohort study

**DOI:** 10.1186/1471-2318-14-111

**Published:** 2014-10-16

**Authors:** Myung Ki, Sujin Baek, Young-duk Yun, Namhoon Kim, Martin Hyde, Baegju Na

**Affiliations:** Department of Preventive Medicine, School of Medicine, Eulji University, Daejeon, Korea; Institute for Health Insurance Policy Research, National Health Insurance Service, Seoul, Korea; Department of Internal Medicine, College of Medicine, Korea University, Seoul, Korea; Stress Research Institute, Stockholm University, Stockholm, Sweden; Department of Preventive Medicine, College of Medicine, Konyang University, Konyang Univ. Gwanjeo Campus, Gasuwon-dong, Seo-gu, Daejeon, 302-833 Korea

**Keywords:** Type 2 diabetes, Diabetes management among older patients, Hospitalization, Glycemic control, Risk factors

## Abstract

**Background:**

Age-related differences in diabetes outcomes are important both for clinical and policy considerations. To clarify the basis of such differences, we investigated patterns of associations for age in relation to hospitalization and glycemic control and examined the role of other factors.

**Methods:**

4471 patients with diabetes aged 40–79 years were drawn from a retrospectively retrieved National Health Insurance Cohort. Using logistic regression, risk factors measured over the two years (2007–2008) were examined for their associations with hospitalization and poor glycemic control during the last year (2009) of follow-up.

**Results:**

Compared to the middle-aged patients, older patients were more likely to have been hospitalized (Adjusted odds ratio (OR_adjusted_) = 1.97(95% CI = 1.28, 3.04) for the oldest group (ages 70–79) vs youngest group (ages 40–49)) but less likely to have poor glycemic control (OR_adjusted_ = 0.45 (95% CI = 0.37, 0.56) for the oldest group vs youngest group). Older patients were also less likely to be obese but had more complications, longer duration of diabetes, lower continuity of care, and higher blood pressure and total cholesterol level. The pattern of associations for hospitalization and glycemic control was not uniform across the risk factors, sharing only a few common factors such as the duration of diabetes and blood pressure. In general, poor glycemic control was affected predominantly by metabolic management, while hospitalization was strongly related to functional status (i.e., number of complications) and care quality measures (i.e., continuity of care).

**Conclusion:**

Hospitalization was higher among the older diabetic patients, despite better glycemic control. Factors were differently associated with the two diabetes-related outcomes, providing more comprehensive risk profiles for hospitalization. The co-existence of improved glycemic control and increased hospitalization among older diabetic patients suggests an extension of a geriatric evaluation to wider functional and comorbidity status.

## Background

The recent epidemic of type 2 diabetes is a global concern [1, 2]. The rise in the prevalence of diabetes has become increasingly alarming, as it has coincided with population aging. South Korea (hereafter Korea) has one of the fastest aging population in the world and this has been accompanied by a surge of diabetes among the older population. According to a representative Korean national survey [3], the standardized prevalence of diabetes among adults aged over 30 increased from 8.9% to 10.1% between 2001 and 2010, while the corresponding figure for those aged 65 or older was 16.6% to 22.7%.

This is a major concern as older diabetic patients are known to have more complications [4], and a higher rate of hospitalization [5–7], and mortality [8]. However, the majority of studies, both those using community and patients based samples [9–13] including Korean studies [3, 14], consistently have shown a clear inverse relationship between age and glycemic control among diabetic patients, though some studies [15, 16] only observed better glycemic control among the oldest-old age group. Overall, this suggests that better glycemic control and other adverse consequences co-exist amongst older diabetic patients. To date, research has focused on such outcomes separately; only a few previous studies [9, 11, 17] have pointed out the inverse association between age and glycemic control versus other important diabetes-related outcomes and none of them has actually examined the associations.

Furthermore, there is some evidence that the relationship between age and better glycemic control may be simply attributed to other characteristics such as BMI (body mass index) [18] and duration of diabetes [19]. Older patients with diabetes tended to be leaner than younger patients, and so this may explain their better glycemic control. Similarly, some studies have shown that age affects glycemic control mainly through the interaction with duration of disease [20], others have only found this effect for those aged 80 and over [19], while other studies found persistent effects of age even after consideration of duration of diabetes [11, 13, 21]. Thus, to clarify these age-related associations, we investigated whether age predicts a greater likelihood of hospitalization and better glycemic control in older patients with type 2 diabetes and, if so, whether the association is independent of other covariates.

Diabetes is a major source of potentially avoidable hospitalization. It is broadly described as an ambulatory care-sensitive condition, for which the provision of quality out-patient care may play a critical role [22]. However, the direct cost of diabetes attached to in-patient care is considerable and on the rise [23] and thus the identification of factors linked to an increased risk of hospitalization may enable considerable financial savings. This is particularly important in the Korean context, where the gap in diabetes management is high-lighted by the very high rates of hospitalization relative to the moderate prevalence of diabetes [24]. Consequently, there has been a growing demand for research to examine the risk factors for increased hospitalization [6, 25]. To assess the prevalence of hospitalization, we investigated measures for quality of diabetes care such as continuity of care, defined as the provision of diabetes care by a usual provider for a period [26], in relation to two patient outcomes (i.e., hospitalization and glycemic control).

Based on a large nationally representative sample linked to clinical data, we aimed to address whether 1) the patterns of age-related associations differ between hospitalization and glycemic control, 2) the age-related associations are independent of other common characteristics of patients with diabetes, and 3) a set of risk factors from various areas are differently associated with hospitalization and glycemic control in direction and magnitude.

## Methods

### Study population

For the current study, 4471 patients diagnosed with diabetes were selected from the Korean National Health Insurance claims data. Korean National Health Insurance is a compulsory enrolment scheme and covers the whole population. Data for 989,887 people from the Korean National Health Insurance were retrospectively collected from January 2001 to December 2009 (Korean National Health Insurance Cohort). The full details of the data construction procedures are described elsewhere [27]. Briefly, the data were drawn based on eight stratification variables (i.e., gender, age, occupation, residential area, types of health insurance, level of premium, mortality and regular health check-up) to proportionately represent 2% of the Korean population and included a variety of information on medical records and laboratory tests.

Several inclusion criteria were applied to deliver the sample used in the current study. Firstly, type 2 diabetic patients aged between 40 and 79 in 2007 were identified from the cohort (n = 60397), via a primary or a secondary diagnosis from insurance claims data, according to the Korean coding system based on ICD 10. Those aged 80 and over were excluded, due to the survival effects often observed for the extremely old age group [16]. Secondly, of those, patients who had been diagnosed at 2001 or who were newly diagnosed between 2002 and 2006 were retained as a baseline sample and followed-up from 2007 to 2009. We excluded those who were newly diagnosed with diabetes after 2007 due to the large variation seen in early years of the disease. Thirdly, patients who made outpatient visits for diabetes less than four times between 2007 and 2008 were also excluded to provide robust measures for some estimates and to assure diagnosis validity. Fourthly, data were further restricted to allow linkage with health examination records including laboratory tests, which were taken as part of the regular national health examination depending on varying eligible criteria (i.e., age and gender). This left 4471 type 2 diabetic patients who met these criteria. Ethical approval was obtained from Institutional Research Board at Konyang University and this study conducted through a secondary data analysis without a personal identifier.

### Measures

The two outcome variables, hospitalization and glycemic control indicated by fasting plasma glucose (FPG), were collected in 2009, the last year of the 9 year follow-up period. Hospitalization was defined as occurring when a patient was admitted for more than one day for a primary or a secondary diagnosis of diabetes, cardiovascular disease, cerebrovascular disease, or renal disease. For glycemic control, a value of FPG greater than 126 mg/dL was taken to indicate hyperglycemia in accordance with the American Diabetic Association (ADA)’s recommendation [28]. If there were more than two FPG values within 2009, the last measurement was used.

A range of predictors were mostly measured over 2007–2008, unless otherwise specified. Age at 2007 was categorized into four groups (ages 40–49, 50–59, 60–69 and 70–79). These groups were aggregated into two groups (ages 40–59 and 60–79) for some analyses. Predictors from the health examination records (i.e., FPG, blood pressure, total cholesterol and BMI) were drawn from 2007 or 2008. When data from more than one laboratory were available, the data collected closest to the 31st December 2007 were selected. Binary variables for hypertension and hypercholesterolemia were defined according to recent ADA and National Cholesterol Education Program Expert Panel recommendation [28, 29]respectively: whether they reached levels of systolic blood pressure (SBP) ≥ 140 mmHg or diastolic blood pressure (DBP) ≥ 90 mmHg and total serum cholesterol ≥240 mg/dL. BMI (kg/m^2^) was calculated using height and weight and categorized into three groups; normal (<25), overweight (25–29.9), and obese (≥30). Prior hospitalization between 2007 and 2008 and prior glycemic control were defined for the same categories as defined above. FPG values from two different years were at least six months apart to represent glycemic control over a different period. Other clinical characteristics at 2008 included duration of diabetes (three categories; 3–5, 6–8 and ≥9 yrs) and number of complications (three categories; 0, 1–2 and ≥3). Diabetic complications were counted, depending on the presence of six categories of micro-vascular (i.e., nephropathy, retinopathy, and neuropathy) and macro-vascular complications (i.e., peripheral vascular disease, cerebrovascular disease, and cardiovascular disease). Health service utilization measures included types of main medical institution attended (five categories; public health centre, clinic, hospital, general hospital and tertiary hospital) and continuity of care measured with continuity of care index (COCI) to assess levels of continuity of the physician-patient relationship [25, 30].The equation for COCI is:


where N = total number of visits, n_j_ = number of visits to provider j, and M = total number of providers. COCI measured over two years (2007–2008) ranged between 0 and 1 (the higher the value the higher the continuity) and was dichotomized into two groups; having been seen always by the same physician (COCI = 1) vs not always (COCI < 1). The cost of the Korean national health insurance premium was calculated based on an individual’s financial status, which was transformed into quartile as a marker for socioeconomic status.

### Statistical analysis

We first conducted simple bivariate analyses to assess the associations of age and covariates with hospitalization and glycemic control using Chi-square tests. Then, multivariate associations were assessed using logistic regression. We undertook two models fitted with different adjustments for covariates to evaluate the pattern of associations of various risk factors with hospitalization and glycemic control. Firstly, Model 1 was fitted for each predictor with only a basic adjustment for gender and age. Secondly, additional adjustments were made for all other variables; prior hospitalization, prior glycemic control, hypertension, hypercholesterolemia, BMI, duration of diabetes, number of complications, types of main medical institution attended, continuity of care, and level of insurance premium (Model 2). To assess the independent association of age with hospitalization and glycemic control, the analyses were repeated on the basic model (i.e., gender and age only model) with separate adjustments for each predictor and which was then compared to the magnitude of the association in the basic model. As a further check, we tested interactions between age and each predictor to see whether the associations between age and hospitalization and glycemic control depended on the level of other variables. For a graphical illustration of the relationship, adjusted prevalence was presented. Adjusted prevalence was calculated by applying the parameter estimates to the mean values of variables, based on the logistic regression model with age and each predictor (as one combined variable, *x*_1_) and gender (*x*_2_). *g*th stratum of *x*_1_ (probability *P*_*g*_(*x*_1_)) can be written;


Apart from the calculation of adjusted prevalence which was done manually, analyses were performed using SAS 9.1 for Windows.

## Results

Among a total of 4471 patients with diabetes followed for 9 years, about half (48.8%) of participants were aged ≥60 and 46% were female (Table 1). Compared to the middle-aged group, the older group had relatively better glucose management both in 2007 and 2009 and a lower BMI in 2007. However, the group had greater rates of hospitalization in both years and higher BP and total cholesterol levels. A more frequent diabetes-related hospitalization and complications, longer duration of diabetes and lower continuity of care were seen among the older patients.

**Table 1:** **Sample characteristics** ^*^ **of middle-aged (n = 2291, 51.2%) and older (n = 2180, 48.8%) patients with diabetes**

	Middle-aged (40–59)	Older (60–79)		Total
	N (%)	N (%)	p-value^§^	N (%)
Gender				
Male	1515(66.1)	1176(54.0)		2691(60.2)
Female	776(33.9)	1004(46.0)	<0.001	1780(39.8)
Hospitalization				
No	2055(89.7)	1844(84.6)		3899(87.2)
Yes	236(10.3)	336(15.4)	<0.001	572(12.8)
Reasons for hospitalization				
Diabetes	98(4.2)	153(7.0)		251(5.6)
Cardiovascular disease	76(3.3)	96(4.4)		172(3.8)
Cerebrovascular disease	46(2.0)	60(2.8)		106(2.4)
Renal disease	16(0.7)	27(1.2)	<0.001	43(1.0)
Glycemic control				
Controlled	925(40.4)	1132(51.9)		2057(46.0)
Uncontrolled(≥126 mg/dL)	1366(59.6)	1048(48.1)	<0.001	2414(54.0)
Prior hospitalization				
No	1909(83.3)	1660(76.1)		3569(79.8)
Hospitalized	382(16.7)	520(23.9)	<0.001	902(20.2)
Prior glycemic control				
Controlled	995(43.4)	1121(51.4)		2116(47.3)
Uncontrolled(≥126 mg/dL)	1296(56.6)	1059(48.6)	<0.001	2355(52.7)
Hypertension				
Normal	1738(75.9)	1476(67.7)		3214(71.9)
Hypertensive^†^	553(24.2)	704(32.3)	<0.001	1257(28.1)
Total cholesterol level				
Desirable	2050(89.5)	1935(88.7)		3985(89.1)
High(≥240 mg/dL)	241(10.6)	245(11.3)	0.44	486(10.9)
BMI, kg/m^2^				
Normal(<25)	1257(54.9)	1230(56.4)		2487(55.6)
Overweight(25–29.9)	897(39.2)	846(38.8)		1743(40.0)
Obese(≥30)	137(6.0)	104(4.8)	0.17	241(5.4)
Duration of diabetes				
3-5 yrs	861(37.6)	607(27.9)		1468(32.8)
6-8 yrs	608(26.6)	529(24.3)		1137(25.4)
≥9 yrs	822(35.9)	1044(47.9)	<0.001	1866(41.7)
Number of complications				
No	850(37.1)	607(27.8)		1457(32.6)
1-2	1200(52.4)	1212(55.6)		2412(54.0)
≥3	241(10.5)	361(16.5)	<0.001	602(13.5)
Continuity of care^‡^				
High	1345(58.7)	998(45.8)		2343(52.4)
Low	946(41.3)	1182(54.2)	<0.001	2128(47.6)
Types of main medical institution attended				
Tertiary hospital	182(7.9)	158(7.2)		340(7.6)
General hospital	358(15.6)	250(11.5)		608(13.6)
Hospital	128(5.6)	92(4.2)		220(4.9)
Clinic	1534(67.0)	1458(66.9)		2992(66.9)
Public health centre	89(3.9)	222(10.2)	<0.01	311(7.0)
Level of health insurance premium				
Lowest quartile	447(19.5)	484(22.2)		931(20.8)
Second quartile	420(18.3)	399(18.3)		819(18.3)
Third quartile	649(28.3)	556(25.5)		1205(27.0)
Highest quartile	775(33.8)	741(34.0)	0.07	1516(33.9)

^*^Hospitalization and glycemic control were measured at 2009, while other measures including prior hospitalization and prior glycemic control were measured between 2007 and 2008.

^†^SBP ≥ 140 mmHg or DBP ≥ 90 mmHg.

^‡^High, if Continuity of Care Index = 1 and low otherwise.

^§^p-value was obtained from Chi-square test.

The bivariate relationships between predictors and both outcomes are shown in Table 2. Female patients were less likely to have poor glycemic control. Age was the only factor that showed opposing and statistically significant patterns of association with hospitalization (9.6% for the youngest group vs 16.8% for the oldest group, p < 0.001) and poor glycemic control (63.7% for the youngest group vs 43.7% for the oldest group, p < 0.001). The pattern of association between age and both hospitalization and controlled glucose was gradual across age groups. Prior hospitalization and prior poor glycemic control strongly affected the recurrence of each outcome but with no cross-associations. For example, 23.1% of those with a prior history of hospitalization had been readmitted in two years later, while this was true for only 10.2% of those without a prior history. Hypertension and high total cholesterol level were unfavourably associated with better glycemic control. Compared to those with a short duration of diabetes, those with a long duration of diabetes had both higher rates of hospitalization and more frequent poor glycemic control. A greater number of complications and poor continuity of care predicted frequent hospitalization but not glycemic control. No statistical associations were seen for BMI, types of main medical institution attended, and the amount of health insurance premium.

**Table 2:** **Prevalence of hospitalization and poor glycemic control (2009) among diabetic patients by socio-demographic and clinical factors (2007–2008)**

	Hospitalization (%)	p-value ^‡^	Poor glycemic control (%)	p-value ^‡^
Gender				
Male	12.2		55.7	
Female	13.8	0.11	51.5	0.006
Age groups				
40-49	9.6		63.7	
50-59	11.3		58.0	
60-69	13.5		51.2	
70-79	16.8	<0.001	43.7	<0.001
Prior hospitalization				
No	10.2		53.8	
Hospitalized	23.1	<0.001	56.2	0.42
Prior glycemic control				
Controlled	11.9		37.9	
Uncontrolled (≥126 mg/dL)	13.7	0.07	68.4	<0.001
Hypertension^*^				
Normal	11.2		53.1	
Hypertensive	14.5	0.0025	56.3	0.03
Total cholesterol level				
Desirable	12.6		53.4	
High(≥240 mg/dL)	14.6	0.22	58.4	0.02
BMI, kg/m^2^				
Normal(<25)	12.9		53.9	
Overweight(25–29.9)	12.3		54.1	
Obese(≥30)	15.2	0.42	53.9	0.99
Duration of diabetes				
3-5 yrs	10.0		48.4	
6-8 yrs	11.3		53.1	
≥9 yrs	15.9	<0.001	58.9	<0.001
Number of complications				
No	9.1		52.8	
1-2	13.1		53.9	
≥3	20.7	<0.001	54.6	0.76
Continuity of care^†^				
High	10.6		52.9	
Low	15.2	<0.001	55.2	0.11
Types of main medical institution attended				
Tertiary hospital	14.1		46.6	
General hospital	13.8		52.6	
Hospital	18.2		55.3	
Clinic	12.2		55.1	
Public health centre	11.6	0.07	53.4	0.07
Level of health insurance premium				
Lowest quartile	13.0		54.0	
Second quartile	12.3		55.3	
Third quartile	14.0		53.5	
Highest quartile	11.9	0.39	53.7	0.87

^*^SBP ≥ 140 mmHg or DBP ≥ 90 mmHg.

^†^High, if Continuity of Care Index = 1 and low otherwise.

^‡^p-value was obtained from Chi-square test.

In the logistic regression analyses (Table 3), older age was associated negatively with hospitalization and positively with glycemic control. To illustrate, compared with the 40–49 age group, the 70–79 age group had an increased likelihood of hospitalization (OR = 1.97, 95% CI = 1.28, 3.04), but a decreased likelihood of poor glycemic control (OR = 0.45, 95% CI = 0.37, 0.56). These associations remained significant even with additional adjustments for covariates, though a large reduction was observed in the magnitude of the association between age and hospitalization. Prior hospitalization and prior poor glycemic control were strong predictors for later hospitalization and later poor glycemic control respectively, but not for the other. Hypertension and duration of diabetes were commonly related to both outcomes. For example, a longer duration of diabetes was associated with higher risks relative to a shorter duration of diabetes, shown by ORs for ≥9 yrs vs 3-5 yrs of 1.83 (95% CI = 1.36-2.43) for hospitalization and of 1.50 (95% CI = 1.31, 1.73) for poor glycemiccontrol. Adjusting for covariates only slightly reduced the magnitude of associations. Patterns of association were similar for the number of complications and continuity of care with strong associations only with hospitalizations. Hypercholesterolemia only showed associations with poor glycemic control and a substantial reduction in the magnitude of association was observed after adjustment. There was no evidence that BMI was associated with hospitalization and glycemic control.

**Table 3:** **Associations** ^*^ **(Odds Ratio, 95% confidence interval) of age and other predictors (2007–2008) with hospitalization and poor glycemic control (2009)**

	Hospitalization		Poor glycemic control	
	Model 1	Model 2	Model 1	Model 2
	OR(CI)	OR(CI)	OR(CI)	OR(CI)
Age groups				
40-49	Reference	-	-	-
50-59	1.34(0.98, 1.83)	1.09(0.77, 1.54)	0.74(0.63, 0.88)	0.85(0.70, 1.05)
60-69	1.70(1.24, 2.33)	1.21(0.91, 1.60)	0.57(0.48, 0.67)	0.65(0.52, 0.80)
70-79	1.97(1.28, 3.04)	1.47(1.03, 2.12)	0.45(0.37, 0.56)	0.48(0.38, 0.60)
Prior hospitalization				
No	Reference	-	-	-
Hospitalized	2.83(2.07, 3.85)	2.04(1.49, 2.78)	1.08(0.88, 1.32)	1.16(0.93, 1.43)
Prior glycemic control				
Controlled	Reference	-	-	-
Uncontrolled (≥126 mg/dL)	1.27(0.97, 1.64)	1.25(0.96, 1.63)	3.49(3.08, 3.95)	3.33(2.94, 3.70)
Hypertension^†^				
Normal	Reference	-	-	-
Hypertensive	1.32(1.07, 1.63)	1.30(1.05, 1.60)	1.15(1.00, 1.32)	1.15(1.00, 1.33)
Total cholesterol level				
Desirable	Reference	-	-	-
High (≥240 mg/dL)	1.28(0.83, 1.93)	1.27(0.83, 1.94)	1.24(1.03, 1.49)	1.21(1.01, 1.46)
BMI, kg/m^2^				
Normal(<25)	Reference	-	-	-
Overweight(25–29.9)	1.31(0.80, 2.16)	1.39 (0.83, 2.32)	1.02(0.77, 1.34)	1.04(0.79, 1.39)
Obese(≥30)	1.39(0.83, 2.31)	1.43(0.86, 2.38)	1.03(0.78, 1.36)	1.09(0.82, 1.45)
Duration of diabetes				
3-5 yrs	Reference	-	-	-
6-8 yrs	1.59(1.16, 2.17)	1.45(1.05, 2.00)	1.24(1.06, 1.44)	1.17(0.99, 1.37)
≥9 yrs	1.83(1.36 2.43)	1.62(1.21, 2.18)	1.50(1.31, 1.73)	1.39(1.19, 1.61)
Number of complications				
No	Reference	-	-	-
1-2	1.85(1.39, 2.44)	1.43(1.04, 1.92)	0.94(0.78, 1.14)	0.93(0.76, 1.14)
≥3	3.52(2.41, 5.12)	2.27(1.49, 3.33)	0.93(0.77, 1.13)	0.88(0.71, 1.10)
Continuity of care^‡^				
High	Reference	-	-	-
Low	1.86(1.44, 2.38)	1.43(1.10, 1.89)	1.11(0.97, 1.26)	1.06(0.93, 1.22)

^*^Associations were estimated using logistic regression. Model 1was adjusted for gender and age. Model 2 was adjusted for types of main medical institution attended and level of health insurance premium as well as the covariates listed in the table.

^†^SBP ≥ 140 mmHg or DBP ≥ 90 mmHg.

^‡^High, if Continuity of Care Index = 1 and low otherwise.

**Figure 1:**
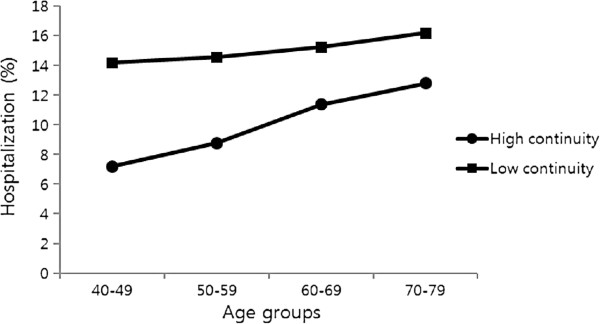
**Adjusted prevalence** ^**†**^ **of hospitalization(2009) by age groups and continuity of care (2007–2008).** ^†^All prevalence estimates were obtained from multivariate models adjusted for gender.

When testing an independent association of age for the two outcomes, ORs for age was attenuated slightly with a simultaneous adjustment for each predictor, suggesting that age has an independent association. The largest change in the OR for age was observed following the adjustment for continuity of care in the association with hospitalization (13.8% reduction in the OR between 70–79 vs 40–49 from 1.97 to 1.72) (Table 4). Finally, there was little evidence of a moderating effect of other predictors on the associations between age and two outcome variables, except for continuity of care for hospitalization (*p* for interaction <0.001); the association between age and hospi-talization was stronger for those with low continuity of care than those with high continuity of care (Figure 1).

**Table 4:** **Changes in Odds Ratio** ^*^ **(95% confidence interval) of age for hospitalization and poor glycemic control with introduction of covariates**

	Age and sex alone	Prior hospitalization	Prior glycemic control	Hypertension ^†^	Total cholesterol level	BMI, kg/m ^2^	Duration of diabetes	Number of complications	Continuity of care ^‡^
	OR(CI)	OR(CI)	OR(CI)	OR(CI)	OR(CI)	OR(CI)	OR(CI)	OR(CI)	OR(CI)
Hospitalization									
Age groups									
40-49	Reference	-	-	-	-	-	-	-	-
50-59	1.34(0.98, 1.83)	1.31(0.95, 1.80)	1.33(0.97, 1.83)	1.32(0.96, 1.82)	1.33(0.97, 1.83)	1.34(0.98, 1.83)	1.30(0.95, 1.78)	1.28(0.93, 1.75)	1.31(0.96, 1.81)
60-69	1.70(1.24, 2.33)	1.61(1.17, 2.21)	1.69(1.23, 2.33)	1.63(1.20, 2.26)	1.68(1.22, 2.31)	1.69(1.24, 2.33)	1.59(1.16, 2.18)	1.55(1.12, 2.13)	1.57(1.14, 2.16)
70-79	1.97(1.28, 3.04)	1.85(1.19, 2.86)	2.00(1.30, 3.11)	1.95(1.24, 2.97)	1.96(1.27, 3.04)	1.98(1.28, 3.06)	1.76(1.14, 2.72)	1.74(1.12, 2.69)	1.72(1.11, 2.67)
Poor glycemic control									
Age groups									
40-49	Reference	-	-	-	-	-	-	-	-
50-59	0.74(0.63, 0.88)	0.74(0.63, 0.88)	0.76(0.64, 0.91)	0.75(0.64, 0.90)	0.73(0.62, 0.86)	0.74(0.63, 0.88)	0.72(0.60, 0.85)	0.74(0.63, 0.88)	0.73(0.62, 0.87)
60-69	0.57(0.48, 0.67)	0.57(0.48, 0.67)	0.59(0.50, 0.70)	0.59(0.50, 0.69)	0.54(0.46, 0.65)	0.57(0.48, 0.67)	0.53(0.45, 0.63)	0.57(0.48, 0.67)	0.55(0.47, 0.65)
70-79	0.45(0.37, 0.56)	0.45(0.37, 0.56)	0.52(0.42, 0.65 )	0.47(0.39, 0.59)	0.43(0.35, 0.54)	0.45(0.37, 0.55)	0.40(0.33, 0.50)	0.45(0.36, 0.55)	0.43(0.35, 0.53)

^*^Odds ratios were estimated using logistic regression. Each covariate was added to a model with age and sex.

^†^SBP ≥ 140 mmHg or DBP ≥ 90 mmHg.

^‡^High, if Continuity of Care Index = 1 and low otherwise.

## Discussion

About half (51.9%) of the patients with diabetes achieved the conventional goal of glycemic control (FPG < 126 mg/dL) and better glycemic control was observed among the older groups. Age was associated with both hospitalization and poor glycemic control, but in opposite directions. The trend remained after adjustment for covariates, suggesting that age is a major independent predictor both for a greater risk of hospitalization and for better glycemic control. No interactions were observed for any combination of age and other covariates, except for age and continuity of care for hospitalization. Factors were differently associated with increased hospitalization and poor glycemic control. For example, metabolic control, such as the levels of FPG and total cholesterol, was an important predictor for poor glycemic control, while prior hospitalization, number of complications, and continuity of care only predicted a higher risk of hospitalization. Hypertension and a longer duration of diabetes were unfavourably related to both outcomes, but no effect was observed for BMI.

### Methodological consideration

This study has several strengths; i) it is nationally representative with diabetic patients from various types of hospitals and clinics, while other studies have focused solely one level of health delivery (i.e., only hospital patients [5, 31, 32] or only primary care patients [7]), or narrowly targeted patient recruitment from a few clinics [31, 32]; ii) it included a range of clinical variables including laboratory data, which have rarely been investigated concurrently, primarily because of the limited availability of such data; and iii) substantial adjustments were made for possible confounding factors such as BMI and continuity of care, which seem to involve age-related differences in diabetes outcomes.

We also note some limitations. Though HbA1c has been acknowledged as a primary test for monitoring of glycemic control, a single measure of FPG was used for an assessment of glycemic control, as HbA1c was not a routine measure in health examinations by Korean National Health Insurance. Even with this limitation, FPG could provide a certain value on the grounds that day-to-day management of diabetic patients including self-monitoring is frequently based on glucose level and some studies support a direct correlation between FPG and HbA1c [33]. Second, whilst the diagnosis of diabetes using insurance claims data can be prone to some inaccuracy, this may not be a major issue in the current study, as we included patients only when they provided health records of diabetes care for at least four (for participants who included in 2006) and to nine (for those who included in 2001) consecutive years. The reliability of diabetes diagnosis was assured by results from a validity study with Korean National Health Insurance Data, which found diagnostic accuracy of 87.2% for in--patients and 72.3% for out-patients [34]. Thirdly, though the glucose levels of those who were on diabetes medication may have been influenced by types of treatment [11], we did not deal with treatment modality because of the lack of relevant information. As such, we could not examine the possibility, if strict glycemic control among older patients is primarily attributable to treatment choice.

### Interpretation and comparison with previous studies

Tighter glycemic control increased incrementally with age. This was contrary to the association between age and hospitalization which showed a consistent increase with advancing ages, correspondingly to the progressive nature of the disease. There are several possible explanations for these findings; true age effects, confounding effects due to characteristics other than age, or a combination of these. In a prior study with a community sample [16], age was not a main predictor in a multivariate analysis, though the best glycemic control was observed among the oldest group. In contrast, other studies [9–11, 20] have shown that the age effect remained even after adjustments for duration of diabetes, treatment modality, BMI and emotional distress, supporting the true age effects, which was similarly noted in the current study.

One interpretation is that the older diabetic patients are more susceptible to glycemic control, partly as a result of the increased need for higher dosage and intensified medications to control blood glucose [35]. Consequently, blood glucose values amongst older patients are likely to fluctuate over time and at the same time to have a higher risk of hypoglycemia, and thereby result in a lower average of blood glucose level [36]. This indicates that tight glucose control may have little benefit among older diabetic patients. Second, as evidence [37] suggests, early-onset diabetes may represent different phenotype, with greater beta-cell dysfunction, than late-onset. This may offer a potential explanation as to why younger age is associated with poor glycemic control, though we could not confirm whether the onset of diabetes in middle-aged patients is earlier than that of older patients.

We examined the role of covariates for confounding and mediating effect in the associations between age and hospitalization and glycemic control. A simultaneous adjustment between age and each covariate generally resulted in small changes in the age effect. With a large change in the magnitude, continuity of care partially accounts for the association between age and hospitalization. Additionally, a significant interaction was observed between continuity of care and age for hospitalization; there was a more noticeable benefit of continuity of care for hospitalization among the young patients with diabetes, but less benefit among the older patients. Most likely, worsened glycemic control leads to a higher continuity of care among the older patients rather than vice versa. Though the explanation is tentative and requires further confirmation, this carries particular policy implications in Korea, where referrals between specialist and primary care are loosely organized with the shortage of general practitioners. In Korea, the decision of which type of clinic to go for treatment largely depends on the patient’s own choice and they are easily directed towards a discontinuation of coordinated care. Thus, this finding suggests that improving continuity of care may be an effective intervention to respond to gaps in diabetes care in Korea.

We found that risk factors were different for hospita-lization and glycemic control. Metabolic control such as glucose and total cholesterol level predicted glycemic control well but did poorly on hospitalization, while prior hospitalization, number of complications, and continuity of care were associated with hospitalization but not with glycemic control. Particularly, associations with hospitalization were modestly affected by glycemic con-trol (i.e., achieving FPG < 126 mg/dL), not reaching statistical significance (p = 0.08 in multivariate analysis). One possible interpretation would be that, as seen in some previous studies [38, 39], the association with glucose level may be less obvious with non-cardiovascular diseases than that in cardiovascular diseases. To confirm if this is the case, sub-categories of hospitalization in this study may need to be differentiated, which would be worthwhile for a future study. Alternatively, the older diabetic patients have a higher risk of hospitalization due to hypoglycaemia [35], which generates a U-shaped association between glucose level and hospitalization [40], although some studies [7, 31] supported linear or threshold effects. In general, different sets of risk factors partially explain the disparity between a higher hospitalization and a better glycemic control amongst older diabetic patients and suggest a more comprehensive approach to reduce hospitalization. This implies that the current threshold of FPG used to indicate a conventional treatment goal might not be sufficient by itself to identify the risk of hospitalization among older patients with diabetes. Therefore, our findings provide support for the recent guidelines [41], which outlined an individualized approach with the recognition of wide functional heterogeneity and clinical complexity common in older patients with diabetes.

## Conclusion

A higher hospitalization among the older diabetic patients appeared in the presence of better glycemic control. The disparity between increased hospitalization and improved glycemic control highlighted a difficulty in treatment decisions for older diabetic patients. Factors were differently associated with these two outcomes, providing more comprehensive risk profiles for hospitalization. An emphasis on a geriatric evaluation to include functional and comorbidity status should be addressed for the care of older diabetic patients.

## Consent

This study used secondary data without personal identifier, for which no informed consent is necessary.
